# Outcomes of Indication-Based Reconstruction Strategies in a Retrospective Observational Cohort of Patients with Paprosky Type IIC Acetabular Defects

**DOI:** 10.3390/jcm15114220

**Published:** 2026-05-29

**Authors:** Sezer Astan, Orhan Balta, Eyüp Çağatay Zengin, Mehmet Burtaç Eren, Kürşad Aytekin

**Affiliations:** 1 Department of Orthopaedics and Traumatology, Tokat Gaziosmanpasa University Hospital, Tokat 60100, Turkey; drorhanbalta@hotmail.com (O.B.); zengincagatay@hotmail.com (E.Ç.Z.); mehmetburtac@hotmail.com (M.B.E.); 2 Department of Orthopaedics and Traumatology, Faculty of Medicine, Giresun University, Giresun 28200, Turkey

**Keywords:** revision total hip arthroplasty, dual mobility cup, reconstruction cage, Paprosky type IIC acetabular defect, dislocation, implant survivorship

## Abstract

**Background/Objectives:** Paprosky Type IIC acetabular defects encountered during revision total hip arthroplasty (rTHA) present substantial challenges in terms of surgical planning and implant stability. This study evaluates the outcomes of indication-based reconstruction strategies using dual mobility cups (DMC) and reconstruction cages (RC) in patients with Paprosky Type IIC acetabular defects. **Methods:** This retrospective, non-randomized study included 41 patients who underwent revision total hip arthroplasty for Paprosky Type IIC acetabular defects between 2014 and 2023, reflecting an indication-based treatment strategy. Patients were categorized into two groups: the DMC group (*n* = 25) and the RC group (*n* = 16). Clinical evaluation was performed using the Harris Hip Score (HHS), while radiographic assessment focused on the restoration of the hip center of rotation. Complications, revision rates, and implant survivorship were also analyzed. **Results:** Both groups demonstrated significant functional improvement; however, the differences in postoperative HHS were observed between groups (86.1 vs. 74.7; *p* < 0.001). The DMC group also showed a shorter operative time, reduced blood loss, and a shorter hospital stay (*p* < 0.05). Although the dislocation rate was lower in the DMC group (4% vs. 12.5%), the difference was not statistically significant. The overall complication rate was markedly higher in the RC group (68.8% vs. 28.0%; *p* = 0.010). Implant survivorship was high in both groups (92.7%), with no significant difference between them. Mean follow-up duration was 49.9 ± 16.0 months. **Conclusions:** Both dual mobility cups and reconstruction cages can achieve successful outcomes in revision total hip arthroplasty for Paprosky Type IIC acetabular defects. However, the observed differences in perioperative and functional outcomes should be interpreted within the context of indication-based patient selection and do not imply superiority of one reconstruction strategy over the other. Rather, these findings reflect outcomes of reconstruction strategies applied according to defect reconstructability in a real-world clinical setting.

## 1. Introduction

Revision total hip arthroplasty (rTHA) has become one of the most complex and rapidly expanding areas of modern orthopedic practice, driven by the increasing frequency of primary arthroplasties, longer life expectancy, and the expanding indications for arthroplasty in younger patient populations [1]. However, rTHA is a far more challenging surgical procedure than primary arthroplasty due to its technical complexity and the higher rate of associated complications [1].

One of the major challenges in revision surgery is determining the extent of acetabular bone loss and assessing the remaining bone morphology. Acetabular bone defects encountered during revision total hip arthroplasty can be classified using several systems, including the American Academy of Orthopaedic Surgeons (AAOS) classification and the Paprosky classification. Among these, the Paprosky classification is the most widely used in clinical practice due to its ability to guide surgical planning based on defect morphology and remaining bone stock. Within this system, Type IIC defects represent a distinct subgroup characterized by medial wall deficiency with preservation of the superior dome, resulting in a unique biomechanical challenge in achieving stable acetabular reconstruction. In the Paprosky classification, Type IIC defects are characterized by loss of medial wall integrity, less than 3 cm of superior migration, disruption of the Kohler’s line, and combined cavitary–segmental bone loss [2]. In this defect type, the success of surgical reconstruction depends on restoring the anatomical center of hip rotation, achieving primary implant stability, and minimizing the risk of dislocation. However, weakness of the medial wall, insufficient bone stock, and complex acetabular morphology make it challenging to achieve these goals [3,4]. Therefore, conventional hemispherical press-fit cup implants often fail to provide sufficient stability in these patients and require additional structural support solutions [5]. We refer to conventional single-mobility hemispherical cups; however, dual mobility cups—despite sharing a hemispherical shell design—provide enhanced stability through their dual-articulation mechanism and therefore represent a distinct reconstruction option in instability-prone revision cases. To overcome these challenges, RC groups that transfer load through the stronger pelvic structures (ilium and ischium) rather than relying on conventional hemispherical implants have been used for a long time [6]. These systems enhance mechanical stability and provide a favorable environment for graft integration [2]. However, in long-term outcomes, the incidence of complications such as mechanical fatigue, metal fracture, and aseptic loosening limits the effectiveness of cage reconstruction, particularly in younger and more active patients [2]. Regis et al. reported that the survivorship rates of such reconstructions significantly decreased in follow-ups beyond 10 years [7].

In recent years, Dual mobility cups (DMC) have gained prominence due to their biomechanical advantages. Originally described by Bousquet et al. in the 1970s, this design increases the effective femoral head diameter, thereby reducing the risk of impingement and enhancing resistance to instability [8]. Recent systematic reviews have demonstrated that DMC significantly reduce dislocation rates in rTHA cases, while also providing reliable outcomes regarding aseptic loosening [9,10,11]. In particular, the use of DMC in high-risk patient groups is considered an effective biomechanical solution against dislocation [1,9,12,13]. Several studies support the use of DMC in revision procedures involving bone defects where instability risk is high [13,14,15,16].

Although dual-mobility (DMC) components are traditionally classified as bearing systems and reconstruction cages as fixation devices, Paprosky Type IIC defects create a surgical scenario in which the key decision is whether the surgeon should attempt a press-fit acetabular reconstruction (with or without DMC) or choose a RC group-based structural reconstruction. Therefore, in clinical practice these two methods function as alternative reconstruction strategies for the same defect type, and the comparison in this study reflects this real-world decision-making process rather than a bearing-type versus fixation-philosophy comparison.

In the current literature, studies directly comparing dual mobility (DM) systems and RC group applications used in rTHA for Paprosky Type IIC acetabular defects are lacking. Previous research has primarily focused either on the mechanical advantages of RC group in cases of severe bone loss and pelvic instability or on the effectiveness of dual mobility cups in reducing dislocation rates. However, these two approaches have not been directly compared in terms of clinical, radiographic, and functional outcomes in patients with the same defect type and comparable demographics [17,18,19,20]. This gap represents a significant lack of knowledge in determining the optimal reconstruction strategy for Type IIC acetabular defects. Therefore, the aim of this study was to evaluate the clinical and radiographic outcomes of two indication-based reconstruction strategies—dual mobility cups and reconstruction cages—in patients with Paprosky Type IIC acetabular defects within a real-world surgical decision-making framework. The primary research question was whether indication-based use of dual mobility cups or reconstruction cages was associated with differences in functional outcomes, complication rates, and radiographic restoration in patients with Paprosky Type IIC acetabular defects.

## 2. Materials and Methods

This study was designed as a cohort study in which patients who underwent rTHA for Paprosky Type IIC acetabular defects at a tertiary university hospital were retrospectively reviewed. Consecutive patients operated between August 2014 and October 2023 with a minimum clinical and radiographic follow-up of 24 months were included in the study. Data were retrospectively collected using electronic medical records (Enlil Hospital Information Management System, Version V2.19.46 2019118) and archived radiographs (Sectra IDS7, Sectra AB, Linköping, Sweden).

### 2.1. Inclusion Criteria

The acetabular defect status was evaluated on anteroposterior hip radiographs according to the Paprosky classification. All radiographs were independently assessed by two senior revision arthroplasty surgeons, and any disagreement was resolved by consensus. Paprosky Type IIC defects were defined as cases with a deficient medial wall, medial migration of the hip center, preservation of the superior dome, and absence of superior migration exceeding 3 cm. A confirmed intraoperative diagnosis and complete clinical and radiographic follow-up were required for inclusion.

### 2.2. Exclusion Criteria

Patients with Paprosky Type III or higher defects, Type IIA or IIB defects, tumor-related reconstructions, less than 24 months of follow-up, or incomplete clinical or radiographic data were excluded.

### 2.3. Ethical Approval

The study was approved by the institutional ethics committee (Approval No: 25-MOBAEK-349), and all procedures were conducted in accordance with the Declaration of Helsinki.

### 2.4. Data Collection and Study Variables

All patient data were retrospectively collected using electronic medical records and archived radiographs. Aseptic loosening was analyzed both as an indication for revision surgery and as a postoperative outcome; these variables were evaluated separately to avoid conceptual overlap. Demographic variables included age, sex, body mass index (BMI), affected side (right/left), American Society of Anesthesiologists (ASA) score, and Charlson Comorbidity Index (CCI). Perioperative variables included operation time (minutes), intraoperative blood loss (mL), use of bone graft, length of hospital stay (days), and implant (cup) diameter (mm). Estimated intraoperative blood loss was calculated based on suction canister volume and surgical sponges, subtracting irrigation fluids. Operative time (minutes) and estimated intraoperative blood loss (mL) were recorded as perioperative variables and included in the comparative analysis. Recorded postoperative outcomes included dislocation, recurrent dislocation, periprosthetic joint infection, aseptic loosening, heterotopic ossification, nerve injury, superficial infection, deep infection, and re-revision for any reason. No thromboembolic complications, including deep vein thrombosis, were recorded during the study period.

### 2.5. Surgical Technique

Patients were divided into two groups according to the type of acetabular reconstruction performed. Implant selection followed a predefined indication-based surgical algorithm. DMC was preferred when the medial wall defect was reconstructable with graft support and adequate rim stability. Cage reconstruction was selected when medial wall deficiency compromised rim support or when adequate rotational stability of a press-fit cup could not be achieved. Therefore, treatment allocation was not randomized and was based on intraoperative assessment of defect reconstructability and stability. Consequently, the observed differences between groups are likely influenced by underlying differences in defect severity and reconstructability rather than the reconstruction method alone. As a result, patients in the two groups differed in baseline defect characteristics and reconstructability, introducing inherent selection bias and limiting direct comparability between reconstruction strategies. The DMC group consisted of cases in which a dual mobility acetabular component (Polar Cup Dual Mobility System, Smith & Nephew) was used, whereas the RC group included cases reconstructed with a reconstruction cage (Contour Acetabular Ring, Smith & Nephew). All procedures were performed using a posterolateral surgical approach by experienced arthroplasty surgeons from the same team. Surgeries were conducted with the patient in the lateral decubitus position. Of the total cohort, 31 procedures involved isolated acetabular (cup) revision, whereas 10 cases included combined acetabular–femoral revision. Of these, combined acetabular–femoral revisions were performed in 4 patients in the DMC group and 6 patients in the RC group. Statistical adjustment did not demonstrate a significant association between the type of revision and postoperative COR measurements or HHS outcomes; however, this finding should be interpreted cautiously given the limited sample size. During surgery, the existing components were carefully removed, and all fibrotic tissues and particulate debris were excised. The type of acetabular defect was confirmed intraoperatively according to the Paprosky classification, based on direct assessment of medial wall integrity, rim support, and the ability to achieve stable fixation. This intraoperative confirmation was based on surgeon judgment and was not supported by predefined objective criteria or standardized documentation, which may limit the reliability of classification. In the DMC group, the defect area was supported with autograft or allograft bone, and the appropriately sized revision cup was implanted using a press-fit technique and additional screw fixation when necessary. The cup type and size were determined using trial components, and if required, impaction bone grafting with allograft was applied to minimize the defect.

In the RC group, after bone grafting of the defect, the reconstruction cage was positioned onto the ischium and ilium and fixed with cortical screws. A suitable polyethylene liner was then cemented or placed into the cage according to stability.

**Figure 1:**
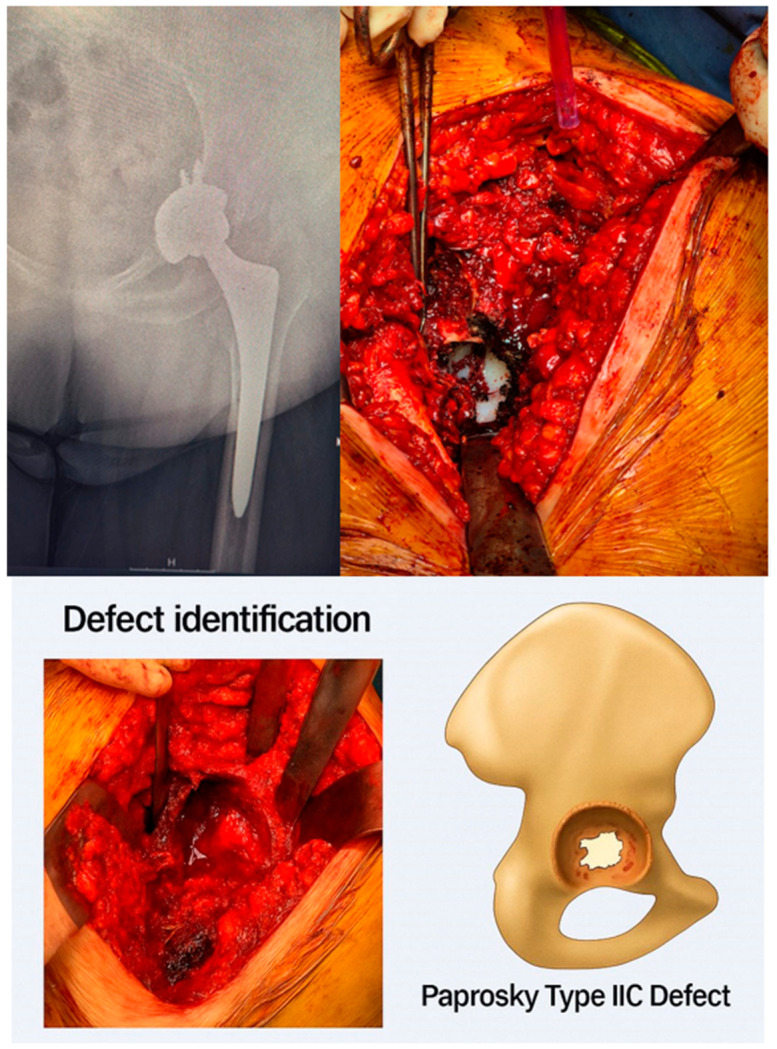
Radiological and intraoperative views of a Paprosky Type IIC acetabular defect.

In the postoperative period, both groups received standard antibiotic prophylaxis and deep vein thrombosis (DVT) prophylaxis. Patients were advised partial weight-bearing for the first six weeks, followed by gradual progression to full weight-bearing as tolerated (Figure 1).

### 2.6. Clinical and Radiographic Evaluation

Clinical outcomes were evaluated using the HHS at the preoperative and final follow-up assessments. Patients were invited for a follow-up visit, which included anteroposterior and axial hip radiographs and a clinical examination for HHS evaluation. A total of 36 patients were evaluated in person, while 5 patients were assessed via structured telephone interview. No formal validation of HHS collection via telephone was performed. Final follow-up was defined as the last available clinical and radiographic assessment performed at a minimum of 24 months postoperatively.

**Figure 2:**
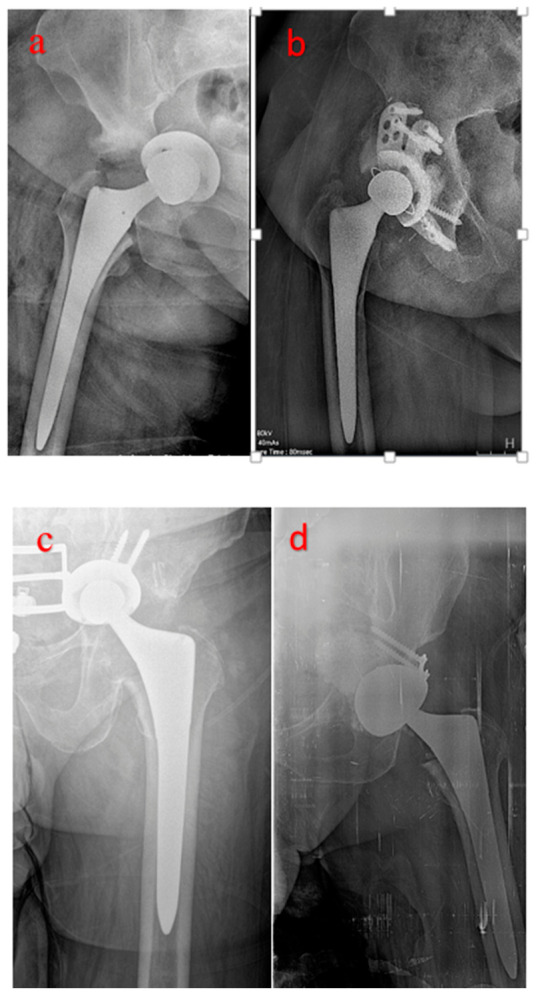
(**a**) Preoperative radiograph showing a Paprosky Type IIC acetabular defect; (**b**) postoperative view following cage reconstruction. (**c**) Preoperative radiograph showing a Paprosky Type IIC acetabular defect; (**d**) postoperative view following impaction bone grafting + dual mobility cup reconstruction. Preoperative radiographs demonstrate characteristic features of Paprosky Type IIC defects, including medial wall deficiency (Kohler’s line disruption), medial migration of the hip center, and preservation of the superior dome. Given the potential for overlap with more advanced defect patterns, some degree of misclassification cannot be excluded.

**Figure 3:**
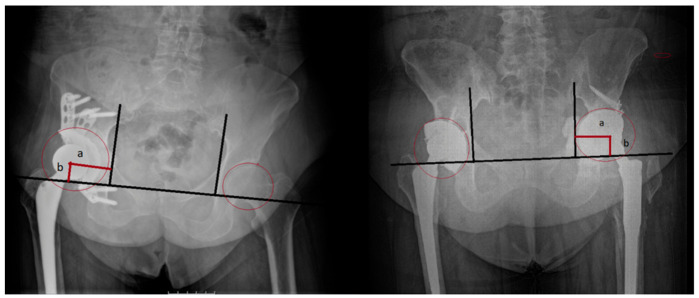
Radiographic illustration showing the vertical and horizontal positions of the center of rotation (COR): (a) vertical position of the COR; (b) horizontal position of the COR.

Radiographic evaluation was performed using anteroposterior pelvic radiographs obtained preoperatively, postoperatively, and at the final follow-up. The horizontal and vertical positions of the hip center of rotation were measured to assess the degree of anatomical restoration. Cup loosening was defined as the presence of circumferential radiolucent lines, cup migration, or fractured fixation screws (Figure 2 and Figure 3).

Implant survival was analyzed using the Kaplan–Meier method, with any revision surgery for any reason considered as the endpoint. Statistical analysis was performed using IBM SPSS Statistics software (version 26.0; IBM Corp., Armonk, NY, USA). Continuous variables were expressed as mean ± standard deviation (SD), and categorical variables as number (*n*) and percentage (%). The normality of data distribution was assessed using the Shapiro–Wilk test. Comparisons of normally distributed continuous variables between the DMC and RC groups were performed using the independent-samples *t*-test, while categorical variables were analyzed using Pearson’s chi-square test or Fisher’s exact test when appropriate. Within-group preoperative–postoperative comparisons of HHS and radiographic measurements were performed using the paired-samples *t*-test. Group differences in operative variables, clinical outcomes, and radiographic parameters were analyzed using appropriate parametric or nonparametric tests. To identify factors associated with complication occurrence and the need for re-revision, variables were entered into a multivariate logistic regression model. Given the limited number of outcome events relative to the number of covariates, the multivariable model may be unstable and prone to overfitting, and therefore should be interpreted cautiously. Age, sex, BMI, operative time, blood loss, and type of reconstruction method (DMC or RC group) were included as covariates. The model’s goodness of fit was assessed using the Hosmer–Lemeshow test, and results were reported as odds ratios (ORs) with 95% confidence intervals (CIs). In addition, differences in implant survivorship between groups were compared using the log-rank test. A two-tailed *p* value of <0.05 was considered statistically significant for all analyses.

## 3. Results

**Figure 4:**
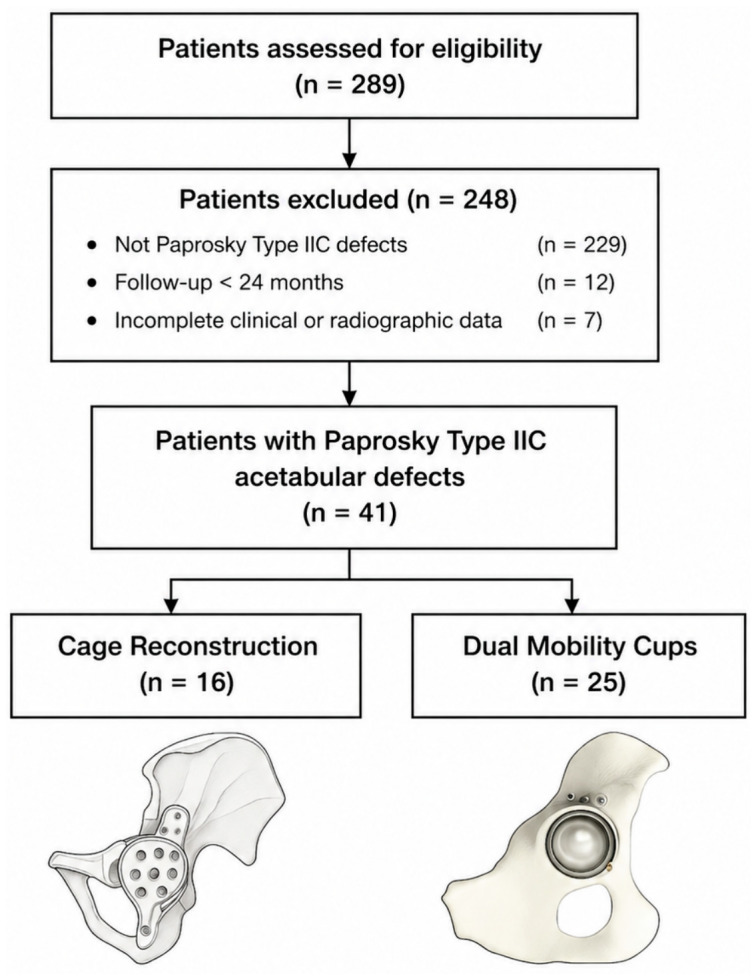
Flowchart of patient selection and group allocation. A total of 289 patients were assessed for eligibility. After applying exclusion criteria, 248 patients were excluded (not Paprosky Type IIC defects, *n* = 229; follow-up < 24 months, *n* = 12; incomplete clinical or radiographic data, *n* = 7). A total of 41 patients with Paprosky Type IIC acetabular defects were included in the study and allocated to the dual mobility cup (DMC) group (*n* = 25) or the reconstruction cage (RC) group (*n* = 16).

A total of 41 patients were included in the study (DMC group, *n* = 25; RC group, *n* = 16) (Figure 4). No cases initially classified as Paprosky Type IIC were reclassified intraoperatively as higher-grade defects. There were no significant differences between the groups regarding sex, operated side, type of revision, implant survival, or the use of bone allograft (*p* > 0.05). The most common indication for acetabular revision was aseptic loosening (63.4%), with no difference between the groups. Complications were more frequent in the RC group than in the DMC group, with a significantly higher overall complication rate in the RC group (Table 1).

A total of 41 patients were included in the study (25 in the DMC group and 16 in the RC group). Baseline demographic characteristics were largely comparable between groups, although time to revision, preoperative HHS, and selected radiographic parameters differed (Table 2). Overall, differences in perioperative and postoperative functional outcomes were observed between the groups, with observed numerical differences between groups (Table 2); however, these findings should be interpreted within the context of indication-based treatment allocation. These numerical differences are presented for descriptive purposes only and should not be interpreted as evidence of comparative effectiveness or treatment superiority.

**Figure 5:**
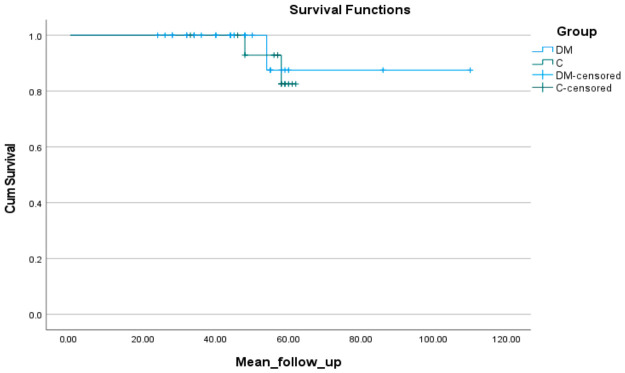
Kaplan–Meier survival curve.

Both groups showed significant improvement in center-of-rotation measurements from the preoperative to postoperative period. Differences in final horizontal and vertical COR distances were observed between groups (Table 2); however, these findings should be interpreted descriptively within the context of indication-based treatment allocation rather than as evidence of comparative advantage. Complication rates were higher in the RC group, whereas implant survivorship did not differ significantly between groups (Table 1, Figure 5). All between-group comparisons in this study should be interpreted as descriptive observations rather than comparative estimates of treatment effect.

Patients with implant failure had undergone a greater number of previous revisions (*p* = 0.005), had higher CCI scores (3.7 ± 0.6 vs. 2.2 ± 0.7; *p* < 0.001), and demonstrated lower postoperative HHS values (70.0 ± 8.7 vs. 82.6 ± 9.0; *p* = 0.025). In addition, infection as the etiology was associated with implant failure (*p* = 0.010).

In univariate logistic regression analysis, the RC group was associated with an increased risk of complications (OR = 5.657; 95% CI: 1.436–22.286; *p* = 0.013). Longer operative time, greater blood loss, and lower postoperative HHS were also associated with complications.

In the multivariable model, postoperative HHS was identified as a factor associated with complications (OR = 0.866; 95% CI: 0.789–0.951; *p* = 0.003); however, this finding should be interpreted cautiously given the limited sample size and low number of events.

Kaplan–Meier analysis showed implant survivorship of 87.5% at 54 months in the DMC group and 82.5% at 58 months in the RC group, with no statistically significant difference between groups (log-rank *p* = 0.816) (Figure 5).

Notably, several baseline differences were observed between the groups, including preoperative HHS, time to revision, and radiographic parameters. These findings indicate the presence of residual confounding and should be considered when interpreting the comparative results. In particular, differences in preoperative HHS, time to revision, and radiographic parameters suggest variation in underlying defect severity between groups. These baseline differences further support the presence of indication bias in treatment allocation.

## 4. Discussion

The main findings of this study were that both dual mobility cups (DMC) and reconstruction cages (RC) provided clinically meaningful improvement in patients undergoing revision total hip arthroplasty for Paprosky Type IIC acetabular defects. Differences in operative time, estimated blood loss, length of hospital stay, postoperative Harris Hip Score (HHS) values, and complication rates were observed between groups; however, these findings should be interpreted within the context of indication-based treatment allocation rather than as evidence of comparative superiority. In addition, both reconstruction strategies improved radiographic restoration of the hip center of rotation. Differences in radiographic measurements were observed between groups; however, these findings should be interpreted cautiously within the context of indication-based treatment allocation. Despite these differences, implant survivorship was similarly high in both groups during mid-term follow-up. Because treatment allocation was based on defect characteristics and intraoperative stability, the groups were inherently non-comparable, limiting causal interpretation of the observed differences. Therefore, the observed differences between groups likely reflect underlying defect severity and reconstructability rather than true differences in implant performance. Accordingly, the present study should be interpreted as an observational analysis of indication-based reconstruction strategies rather than a direct comparative effectiveness study.

This indication-based approach reflects real-world surgical decision-making but introduces selection bias and residual confounding, which limit the ability to draw causal conclusions.

DMC increases the effective femoral head diameter, thereby reducing the risk of impingement and significantly enhancing dislocation resistance by increasing the “jump distance.” This feature provides clinically meaningful benefits, particularly in revision procedures where abductor mechanism weakness and soft tissue imbalance are frequently encountered. In our study, the dislocation rate in the DMC group was 4.0%, which was lower than that observed in the RC group (12.5%). This finding is consistent with the results reported by Kim et al., who demonstrated a significant reduction in dislocation rates with DMC use in isolated cup revisions [1]. This finding is further supported by several reports in the literature. Similarly, Hitz et al. reported a dislocation rate of only 2.9% at a mean follow-up of 8 years in patients treated with cemented DMCs implanted into a trabecular metal shell [21]. Toro-Ibarguen et al. demonstrated that the combination of dual mobility cups cemented into antiprotrusio cages significantly reduced dislocation rates [22].

These findings indicate that, within this indication-based treatment framework, differences in functional outcomes and complication rates were observed between groups; however, these observations should be interpreted descriptively and in light of underlying differences in defect severity and reconstructability rather than as evidence of implant superiority.

Accurate restoration of the hip rotation center is essential for maintaining a physiological abductor lever arm and ensuring balanced load transmission. In our study, restoration of the hip center was observed with both reconstruction methods, and differences in horizontal and vertical center distances were observed between groups; however, these findings should be interpreted cautiously within the context of indication-based treatment allocation. This observation is in line with the multicenter study by Philippot et al., which demonstrated that DMC more effectively restore the hip center and reduce dislocation rates [23]. However, biomechanical parameters such as combined offset are also influenced by the design and revision status of the femoral stem. Because combined offset could not be reliably measured retrospectively, this remains a limitation of the present study. Although stem revision was performed in 10 patients, statistical adjustment demonstrated that stem revision did not significantly influence postoperative hip biomechanics.

Similarly, Li et al. demonstrated that center restoration, achieved through 3D modeling–assisted augment applications, was directly associated with improved functional outcomes [24]. Although differences in functional and complication outcomes were observed between groups, these findings should be interpreted cautiously because treatment allocation was indication-based and the groups differed in baseline reconstructability.

RCs remain an important treatment option in cases of severe bone loss, such as medial wall destruction or pelvic discontinuity. O’Neill et al. and Hipfl et al. reported that while RCs can provide short- to mid-term stability, they are also associated with a considerable rate of complications [2,19]. In our study, the overall complication rate in the RC group was 68.8%, which is consistent with the rates reported in the literature. Chang et al. reported a 32% reoperation rate and an 18% mechanical failure rate in patients treated with RC reconstruction. These findings suggest that RC group-based reconstruction remains a necessary option in more severe and mechanically challenging defects, although such cases may also carry a higher baseline risk of complications independent of implant choice [25].

Regis et al. also emphasized that the long-term survival rates of RC markedly decrease beyond 10 years [26].

In our study, patients who developed complications had significantly longer operative times and greater intraoperative blood loss, while their postoperative HHS values were lower. Multivariable analysis identified low postoperative HHS as a factor associated with complications (OR = 0.866; *p* = 0.003). Multivariable findings should be interpreted cautiously due to the limited sample size and low number of events. This finding indicates that functional recovery plays a key role in reducing the risk of postoperative complications. Similarly, Huten et al. identified high BMI and abductor weakness as risk factors in patients who developed dislocation [27].

Implant survivorship was high in both groups, with no statistically significant difference observed between reconstruction strategies during mid-term follow-up. This finding is consistent with the results of Assi et al. [21] who reported a survival rate exceeding 94% at 14-year follow-up using a combination of a Kerboull plate and cemented dual mobility cup [28].

The present study has several important limitations. Given the limited sample size and the low number of events, the multivariable analysis should be considered exploratory, and the findings should be interpreted cautiously due to potential model instability. First, its retrospective design is inherently subject to indication bias, as reconstruction strategy was determined according to defect characteristics, reconstructability, and intraoperative stability rather than random allocation. As a result, the two groups were non-comparable, and causal inferences regarding the superiority of one technique over another cannot be made. Second, baseline differences in preoperative HHS, vertical center of rotation, and time to revision indicate residual confounding that cannot be adequately adjusted for in this cohort and suggest differences in underlying defect severity and clinical complexity. Third, the accuracy and reliability of Paprosky Type IIC classification represent a major methodological limitation of this study. Although all radiographs were independently evaluated by two experienced surgeons and disagreements were resolved by consensus, interobserver agreement was not formally assessed, and misclassification cannot be excluded. In addition, intraoperative confirmation of defect type was based on surgeon judgment and was not supported by predefined objective criteria or standardized documentation. Given that the entire premise of the study relies on accurate identification of Paprosky Type IIC defects, any potential misclassification may substantially affect the validity and interpretation of the findings. Fourth, the inclusion of both isolated acetabular and combined acetabular–femoral revisions introduced additional heterogeneity, and the impact of concomitant femoral revision could not be fully accounted for in the present analysis. Fifth, variability in grafting techniques, fixation strategies, and liner use further limits comparability between groups, as these technical factors may independently influence both mechanical stability and clinical outcomes. Sixth, a proportion of patients were assessed via telephone rather than in-person clinical evaluation, which may compromise the accuracy and reliability of HHS assessment. This study is among the few that directly compare DMC and RC reconstructions within the same cohort of patients with Paprosky Type IIC defects. However, its retrospective design, limited sample size, and the absence of long-term follow-up data represent the main limitations. Nevertheless, the systematic evaluation of clinical, radiographic, and survivorship parameters constitutes one of the key strengths of this study.

In revision total hip arthroplasty for Paprosky Type IIC acetabular defects, both dual mobility cups and reconstruction cages can be used successfully within an indication-based surgical strategy. In the present cohort, differences in perioperative and functional outcomes were observed between groups; however, these differences should be interpreted in light of indication-based treatment allocation, underlying defect severity, and baseline reconstructability. These findings are consistent with the use of dual mobility cups in appropriately selected Paprosky Type IIC defects with sufficient reconstructability, while reconstruction cages remain an important alternative in cases with greater structural deficiency or inadequate rim support. In conclusion, both dual mobility cups (DMC) and reconstruction cages (RC) can be used successfully in revision total hip arthroplasty for Paprosky Type IIC acetabular defects when selected according to defect reconstructability and intraoperative stability. Although differences in perioperative, functional, and complication-related outcomes were observed between groups, these findings should be interpreted cautiously because treatment allocation was indication-based and the study groups were not randomized. These findings do not indicate superiority of one reconstruction method over the other but rather reflect the outcomes of indication-based surgical decision-making. Importantly, no causal or comparative inference should be drawn from the observed differences, as the two groups were fundamentally non-comparable due to indication-based treatment allocation. Further prospective studies with more homogeneous baseline characteristics are needed to better define the optimal reconstructive strategy for this specific defect pattern.

## Figures and Tables

**Table 1:** Baseline characteristics and categorical variables according to reconstruction group.

Variables				*p* Value
		DMC (*n* = 25)	RC (*n* = 16)	
Gender	Female	18 (72%)	11 (68.8%)	0.823
	Male	7 (28%)	5 (31.3%)	
Operated side	Right	9 (36%)	7 (43.8%)	0.620
	Left	16 (64%)	9 (56.3%)	
Etiology of acetabular revision	Aseptic loosening	15 (60%)	11 (68.8%)	0.794
	Infection	3 (12%)	2 (12.5%)	
	Instability	7 (28%)	3 (18.8%)	
Initial diagnosis	Developmental dysplasia of the hip	8 (32%)	2 (12.5%)	0.336
	Osteoarthritis	11 (44%)	12 (75%)	
	Post-traumatic osteoarthritis	2 (8%)	1 (6.3%)	
	Osteonecrosis of the femoral head	2 (8%)	1 (6.3%)	
	Inflammatory arthritis	2 (8%)	0 (0%)	
Type of revision	Unipolar	9 (36%)	3 (18.8%)	0.236
	Bipolar	16 (64%)	13 (81.3%)	
Implant survivorship	0	24 (96%)	14 (87.5%)	0.550 *
	1	1 (4%)	2 (12.5%)	
Number of previous revision surgeries	0	11 (44%)	3 (18.8%)	0.285
	1	5 (20%)	4 (25%)	
	2	6 (24%)	4 (25%)	
	3	3 (12%)	5 (31.3%)	
Bone allograft used	Yes	17 (68%)	13 (81.3%)	0.478 *
	No	8 (32%)	3 (18.8%)	
Revision for aseptic loosening	No	24 (96%)	13 (81.3%)	0.281 *
	Yes	1 (4%)	3 (18.8%)	

Data are presented as *n* (%). Group comparisons were performed using the Pearson chi-square test or Fisher’s exact test where appropriate. Pearson Chi-Square test was used. *: Fisher Exact test was used. (ab): The same letters as the column indicate statistical insignificance.

**Table 2:** Comparison of perioperative, clinical, and radiographic outcomes between groups.

Variable	DMC (*n* = 25)	RC (*n* = 16)	*p* Value
Demographic variables			
Age (years)	68.68 ± 7.58	69.75 ± 8.68	0.679
BMI (kg/m^2^)	26.49 ± 2.65	27.37 ± 2.88	0.325
Follow-up duration (months)	46.8 ± 19.18	54.88 ± 7.53	0.117
ASA score	2.56 ± 0.51	2.62 ± 0.5	0.689
Charlson Comorbidity Index	2.2 ± 0.82	2.44 ± 0.63	0.329
Perioperative variables			
Time to revision (years)	6.64 ± 3.45	10.19 ± 3.71	0.003
Cup diameter (mm)	53.32 ± 4.96	50.38 ± 3.67	0.048
Length of hospital stay (days)	7.92 ± 3.48	9.94 ± 1.53	0.035
Operative time (min)	103.4 ± 14.49	125.62 ± 20.32	<0.001
Estimated blood loss (mL)	410.8 ± 71.06	620.63 ± 111.56	<0.001
Clinical outcomes			
Preoperative HHS	63.96 ± 8.84	55.75 ± 8.26	0.005
Postoperative HHS	86.08 ± 7.23	74.69 ± 8.32	<0.001
Radiographic outcomes			
Preoperative horizontal COR distance (mm)	35.12 ± 3.48	35.81 ± 2.04	0.477
Postoperative horizontal COR distance (mm)	28.52 ± 4.26	32.5 ± 2.22	0.001
Preoperative vertical COR distance (mm)	44.32 ± 5.44	48.06 ± 1.84	0.012
Postoperative vertical COR distance (mm)	38.12 ± 5.19	42.81 ± 1.56	0.001
Final horizontal COR distance (mm)	29.44 ± 4.15	33.63 ± 2.6	0.001
Final vertical COR distance (mm)	39.12 ± 5.19	43.31 ± 3.46	0.007

Data are presented as mean ± standard deviation. Between-group comparisons were performed using the independent-samples t-test. A *p* value of <0.05 was considered statistically significant.

## Data Availability

Data supporting the findings of this study are available at Tokat Gaziosmanpaşa University Hospital Information Management System, but access to these data is restricted. The data were used under institutional authorization for the current study and are therefore not publicly available. However, the data can be obtained from the corresponding author upon reasonable request and with the permission of Tokat Gaziosmanpaşa University Hospital.

## Abbreviations

DMC	Dual Mobility Cup
RC	Reconstruction cage
rTHA	Revision Total Hip Arthroplasty
HHS	Harris Hip Score
ASA	American Society of Anesthesiologists
CCI	Charlson Comorbidity Index
BMI	Body mass index
